# Developing a Clinical-Grade Cryopreservation Protocol for Human Testicular Tissue and Cells

**DOI:** 10.1155/2013/930962

**Published:** 2013-01-14

**Authors:** Jason Pacchiarotti, Thomas Ramos, Kyle Howerton, Scott Greilach, Karina Zaragoza, Marnie Olmstead, Fariborz Izadyar

**Affiliations:** PrimeGen Biotech LLC, 213 Technology Drive, Irvine, CA 92618, USA

## Abstract

Recent work in preservation of female fertility as well as new information on the nature of spermatogonial stem cells has prompted an investigation into the possibility of an effective clinical-grade procedure for the cryopreservation of testicular cells and/or tissue. Clinical-grade reagents, validated equipment, and protocols consistent with cGTP/cGMP standards were used in developing a procedure suitable for the safe and effective cryopreservation of human testicular cells and tissues. These procedures were designed to be compliant with the relevant FDA regulations. The procedure proved to effectively cryopreserve both testicular cells and tissue. The cryopreservation of testicular tissue was comparable in most aspects we measured to the cryopreservation of isolated cells, except that the viability of the cells from cryopreserved testicular tissue was found to be significantly higher. On the other hand, cryopreservation of cells is preferred for cell analysis, quality control, and sterility testing. This study demonstrates that testicular tissue and cells from sexual reassignment patients can be successfully cryopreserved with a clinical-grade procedure and important cell populations are not only preserved but also enriched by the process. Further studies will determine whether these findings from hormone-treated patients can be generalized to other patients.

## 1. Introduction

Cryopreservation of reproductive cells and/or tissues has become an increasingly important methodology for fertility preservation [1–3]. Success of autologous, cryopreserved ovarian tissue transplantation in patients has shown the ability of transplanted tissue to restore fertility in women and has generated live births [4–6]. Currently, there are no methods for male patients that restore fertility or allow for future generation of new gametes in the event that their fertility is compromised due to testis damage. Cryopreservation of testicular cells and/or tissue prior to any fertility compromising condition or therapy may allow for future cell/tissue transplantation back to the autologous donor so that they may regain the ability to naturally conceive their own biological children [7]. Alternatively, these cells may be used to create new sperm outside the body through germ cell maturation protocols [8]. 

A procedure to preserve male fertility must be proven safe before it can be used in human. Regulations and guidance set up by agencies such as the Food and Drug Administration (FDA) describe the procedures and systems that must be put into place before a product can be deemed safe to use in humans. Investigational techniques for cryopreserving testicular tissue and cells have been tested and reported by several groups [9–12]; however, to our knowledge, a clinical-grade protocol for the cryopreservation of human testicular cells or tissue has not been previously described. All previous studies used protocols noncompliant with current Good Tissue Practice (cGTP) standards, nonclinical-grade reagents, and animal products that made them unfit for clinical use. Additionally, no sterility testing was reported in these studies to ensure the absence of microbial contamination. This study addresses and solves those concerns. The federal cGTP regulations in 21 CFR Part 1271 were used to determine what procedures and systems to put in place. Moreover, at times this study followed the current Good Manufacturing Practices (cGMP) regulations—particularly in terms of equipment validation and documentation. Equipment used in these procedures was validated to ensure proper installation, operation, and performance. Critical points in the procedures were all documented to prove the proper adherence of SOPs. 

Besides creating a clinically applicable procedure, this study directly compares the cryopreservation of cells isolated from fresh human testis with the cryopreservation of whole pieces from the same tissue. Cryopreservation can induce production of ice crystals from the water inside the cells, which can damage the cells' internal structure and cellular membrane—and lead to cell death [12, 13]. Studying the effect of cryopreservation on both cells and tissue will help to determine which method is most suitable and applicable for clinical use. The cells and tissue in this study were cryopreserved in a medium using cryoprotectants to prevent ice crystals from forming, thus improving the ability of the cells to survive freezing and thawing. While some studies have focused on the structural effect of cryopreservation on testicular tissue [13], this study focuses on an analysis of the isolated cells. 

After viability assessment, there are three markers for which the cells will be analyzed that define three important populations of cells in the human testes. First, stage-specific embryonic antigen 4 (SSEA4) has been shown in nonhuman primates and humans to be an effective spermatogonial stem cell (SSC) marker [14, 15]. *In vivo*, SSCs require the support of Sertoli and Leydig cells as they go through spermatogenesis. An effective marker to identify and investigate Leydig cells is the luteinizing hormone receptor (LHR) [16], the second marker used in this study. Finally, VASA (also known as DDX4) is a specific germ cell lineage intracellular marker [17]. All of these cell types, in addition to many other cell types, comprise the heterogeneous mixture of testicular cells and are affected in contrasting ways by cryopreservation of testicular cells or tissue. The differences between cells isolated from fresh tissue will be compared to both the cryopreserved cells and cells from the cryopreserved tissue. Additionally, the differences between the cryopreserved cells and the cells from the cryopreserved tissue will be directly compared. 

The cryopreservation of testicular cells and/or tissues is of particular importance to patients for whom sperm freezing is not an option. Prepubertal patients undergoing radiation and/or chemotherapy are at risk for fertility loss due to the cytotoxic effects of those therapies on the germinal epithelium—where SSCs are located [18, 19]. The direct toxic effects of chemotherapy and radiation exposure on the gonads are generally dose-dependent [20, 21] and the long-term effects of chemotherapy on the testes have not been well characterized. The survival rate among children with cancer has improved over the past several years; close to 80% are expected to survive [22]. Although there are no established options for prepubertal boys who are later found to be infertile, their preserved testicular cells or tissue may potentially be used to restore their fertility [23, 24].

Another population for who this process might be beneficial is men whom have begun the process of sexual reassignment. These patients undergo hormone treatment regimens, which last varying amounts of time and have devastating effects on spermatogenesis in the testes [25]. For this reason, it is usually difficult or impossible for these men to preserve sperm after a critical point in their treatments for use in assisted reproductive techniques. Without sperm, the only option for these men to preserve their fertility may be the preservation of their testicular cells or tissue. This cryopreserved material could then be used in germ cell maturation procedures to produce sperm for use in assisted reproductive techniques.

In this study, testicular tissue from male sexual reassignment patients was used. This was done for two reasons. First, the unavailability of normal human testicular tissue; Second, there are no previous studies for the cryopreservation of testicular cells or tissues from sexual reassignment hormone-treated patients and these patients might directly benefit from this paper. If this process is to be used for these patients, an investigation into the effectiveness of cell and/or tissue cryopreservation is required.

## 2. Materials and Methods

### 2.1. cGTP and cGMP Environment

This study was performed under cGTP guidelines for a product regulated under section 361 of the Public Health Service Act and was compliant with other relevant FDA regulations and guidance at an FDA-registered and inspected tissue processing facility. All critical protocols were performed in a certified clean room. Moreover, protocols were performed using validated equipment and clinical-grade reagents and supplies according to cGMP guidelines. Documentation was followed for quality assurance/quality control and compliance with quality standards and regulations.

### 2.2. Tissue Collection and Testicular Cell Isolation

Sexual reassignment patients included in this study (5) have been treated with hormones for a period of 6–12 months and their age varied between 25 and 40 years. All patients signed an informed consent form with the surgical facility agreeing for their tissue to be used for this study. Testes were surgically removed from the scrotum and washed in sterile cGMP-grade phosphate-buffered saline (PBS, Irvine Scientific, Irvine, CA, USA) before being placed in a sterile bottle of 4°C cGMP-grade PBS (Irvine Scientific). After removal, the testes were shipped overnight in a validated shipper (ThermoSafe, Arlington Heights, IL, USA) between 2°C and 8°C and arrived approximately 24 hours after being removed from the patient.

Upon arrival at the processing facility, the tissue was processed in the certified clean room. The seminiferous tubules were dissected by decapsulating the testes after removal of additional fat and membranes. The tissue was washed in cGMP-grade PBS (Irvine Scientific). A piece of the tissue was cut off, weighed, and placed in a sterile 50 mL conical tube with cGMP-grade PBS (Irvine Scientific) for cell isolation. Other pieces of tissue were cut, weighed, and used for tissue freezing (described below). Fresh or frozen/thawed tissue was dissected by sterile tweezers to smaller strips for enzymatic digestion with Liberase (Roche Applied Science, Indianapolis, IN, USA). Liberase, a cGMP-grade mixture of enzymes, was added to each piece for a final enzymatic digestion concentration of 0.3 units/mL of Collagenase and 1000 units/mL of Thermolysin. The tissue was digested at 37°C on a reciprocating shaker at 110 RPM for 1.75 hours. Undigested tissue was removed from isolated cells by a sterile 100 *μ*m cell strainer (BD, San Jose, CA, USA) before centrifuging the cells at 400 ×g for 5 minutes at 4°C. Cells were resuspended in a mixture of cold cGMP-grade PBS (Irvine Scientific) and 10% human serum albumin (HSA, SeraCare Life Sciences, Milford, MA, USA) and kept at 4°C for further processing.

### 2.3. Cell Count and Viability Assessment

Cells were counted on a validated hemacytometer with the addition of Trypan Blue (Life Technologies, Grand Island, NY, USA) to count the number of dead cells. Each sample was counted twice and an average was taken from the two counts. Viability was calculated by dividing the number of live (viable) cells by the total number of cells counted (live and dead) and displaying the number as a percentage where 100% represents a population of cells that is entirely alive and 0% represents a population that is entirely dead. In addition to Trypan Blue, viability of cells was confirmed using a flow Cytometry based assay by 7AAD staining (see the flow Cytometry analysis section). The number of cells obtained was normalized by the weight of the tissue and expressed as a ratio of viable cells per gram of tissue. 

### 2.4. Cryopreservation

Freshly isolated cells were centrifuged as described above and resuspended in cold cryopreservation media (CM) of 10% HSA (SeraCare Life Sciences), 10%DMSO/1%Dextran (Origen Biomedical, Austin, TX), and cGMP-grade PBS (Irvine Scientific). One mL of cell suspension, containing 3–5 × 10^6^ cells, was pipetted into 1.8 mL cryovials (Nunc, Rochester, NY, USA). Cells were cryopreserved by a validated Kryo-16 Controlled Rate Freezer (Planer, Middlesex, UK). The protocol for the Kryo-16 was as follows: vials were held at 4°C for 10 minutes before being cooled at a rate of −1°C/min to −80°C. The vials were further cooled at a rate of −50°C/min to −120°C. Vials were held at −120°C until they were quickly transferred to a validated MVE TEC 3000 Dewar and stored in the vapor phase of liquid nitrogen at ~−188°C.

Tissue pieces (120 to 500 mg) were cryopreserved in a similar manner. Tissue was placed in a cryovial with 1 mL of cold (4°C) CM and soaked for 30 minutes at 4°C prior to undergoing the cryopreservation procedure.

### 2.5. Flow Cytometry Analysis

Flow cytometry was conducted with a BD FACS Canto (BD) using unstained and secondary-antibody-only stained cells as controls. Cells from freshly isolated and thawed conditions were separately stained with Alexa-488 conjugated anti-human SSEA4 (Ebioscience, San Diego, CA), purified rabbit anti-human LHR (GeneTex, Irvine, CA, USA), and purified rabbit anti-human VASA (Abcam, Cambridge, MA, USA). VASA is an intracellular protein, therefore the cells stained for VASA were first fixed in 4% paraformaldehyde (EMS, Hatfield, PA, USA) overnight and washed in PBS + 0.01% Triton-X. All primary antibody dilutions were optimized at 1 : 200 and staining time was for 30 minutes at 4°C. For stains that required a secondary antibody, cells were first blocked in 10% goat serum for 15 minutes and labeled with a goat anti-rabbit Alexa 488 antibody (Invitrogen, Eugene, OR, USA) at 1 : 500 for 30 minutes at 4°C. All samples included 7AAD (BD Pharmingen, San Diego, CA, USA) to determine and exclude the dead cells during analysis.

For each marker from each sample, the percentage of viable cells positive for that marker was determined. The percentage was then multiplied by the number of total viable cells isolated per gram of tissue to determine how many viable cells positive for each marker were isolated per gram of tissue. These numbers were compared between fresh-tissue cell isolation and either cryopreserved cells or cryopreserved/thawed tissue to determine percent recovery of cells positive for each marker from each type of cryopreservation.

### 2.6. Cell and Tissue Thawing

Cryovials were removed from liquid nitrogen storage and immediately placed in a 37°C water bath. Vials were swirled in the water bath until a small piece of frozen cells remained (~2 minutes). Thawed cells were transferred into a 50 mL conical tube and diluted with 9 mL of 4°C cGMP-grade PBS (Irvine Scientific) and 10% HSA (SeraCare) over the course of several minutes to dilute the CM 1 : 10. The cells were centrifuged as described above and resuspended in PBS (Irvine Scientific) and 10% HSA (SeraCare) for counting as described above. For thawing tissue, the tissue/CM was thawed and the CM was diluted as described above. Instead of centrifugation, the tubes of thawed tissue were held at 0–4°C for 5–10 minutes to allow CM to dilute out of the tissue. Tissue was then transferred to PBS (Irvine Scientific) and placed on ice to await cell isolation. Cell isolation and counting was performed as described above. 

### 2.7. Statistics

Average cell recovery was calculated by dividing the average number of cells after cryopreservation by the average number of cells isolated from fresh tissue. All other averages were calculated by dividing the summation of the values in the category by the sample size. Smith's Statistical Package was used for two sample-student *t*-test for statistical analysis and *P* < 0.05 was considered as significant. Standard error of the mean (SEM) was calculated by dividing the standard deviation by the square root of the sample size. 

### 2.8. Sterility Testing

PBS used for transport of the tissue as well as samples of isolated and/or thawed cells for sterility testing were aseptically collected into sterile 1.8 mL cryovials (Nunc). The vials were shipped to a qualified and CLIA-approved laboratory for sterility testing. Samples were inoculated into Trypticase Soy Broth and Fluid Thioglycollate Medium to test for the growth of yeast, fungi, aerobic, and anaerobic bacteria. Cultures were grown for 14 days. Any detected growth after 14 days was a condition for failure of the sterility test.

## 3. Results

### 3.1. Viability

The viability of the cells is important for determining the effectiveness of the cryopreservation and the condition of the cells after freezing and thawing. The average viability of the cells isolated from fresh testicular tissue was 90.1%. When the same cells were cryopreserved and then thawed, the average viability dropped to 52.4%. When tissue from the same testes was cryopreserved, thawed, and the cells isolated by the same procedure as the fresh tissue, the average viability of the cells was 74.0%—lower than the average viability of cells isolated from fresh tissue by only 16.1% and higher than the average viability of the cryopreserved cells by 21.6% (Table 1). The difference between the viabilities of the cryopreserved cells and the cells from cryopreserved tissue was statistically significant (*P* = 0.0019). This suggests that testicular cells have a better survival rate when frozen as tissue pieces as compared to freezing isolated cells.

### 3.2. Cell Recovery

When cells or tissue are cryopreserved and then thawed, some cells are naturally going to be lost due to cell damage and destruction. These lost cells are not accounted for when performing a simple live-dead count because they are only present as cellular debris. Therefore, comparing just the viability is insufficient. A comparison was made between the number of viable cells initially cryopreserved and the number that remained after thawing. This test was utilized to determine if cryopreserving tissue pieces or cryopreserving isolated cells is a better method: the superior method should yield more viable cells recovered after cryopreservation and thawing. An average of 42.5 × 10^6^ viable cells were isolated per gram of fresh testicular tissue. Upon thawing, an average of 14.0 × 10^6^ of those cells were recovered—a recovery rate of 32.9%. When tissue pieces were cryopreserved, thawed, and enzymatically digested in the same manner as fresh tissue, the number of cells isolated per gram of tissue on average was 37.4% (15.9 × 10^6^ viable cells) of the number of cells isolated per gram of fresh tissue. This indicates that 4.5% additional cells may be recovered, on average, after cryopreservation of tissue pieces. However, this difference was not statistically significant (*P* = 0.78), and it should be noted that only 3 out of the 5 patients showed greater recovery of cells when cryopreserving tissue (Table 2).

### 3.3. Cell Marker Analysis

**Figure 1:**
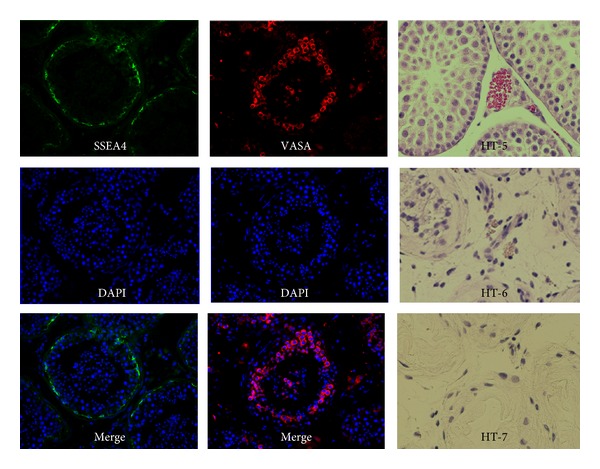
Immunolocalization of cell marker and representative images of testicular cross-sections from estrogen-treated patients. This figure shows localization of SSEA4 and VASA positive and various degrees of spermatogenesis in the human testicular tissue collected from sexual reassignment patients. SSEA4 only stains the cells along the basement membrane of the seminiferous tubules. VASA stains all the germ cells including those along the basement membrane and in the lumen of the seminiferous tubules. Note various degrees of spermatogenesis were found in testes collected from three sexual reassignment patients (HT-5, HT-6, and HT-7).

**Figure 2:**
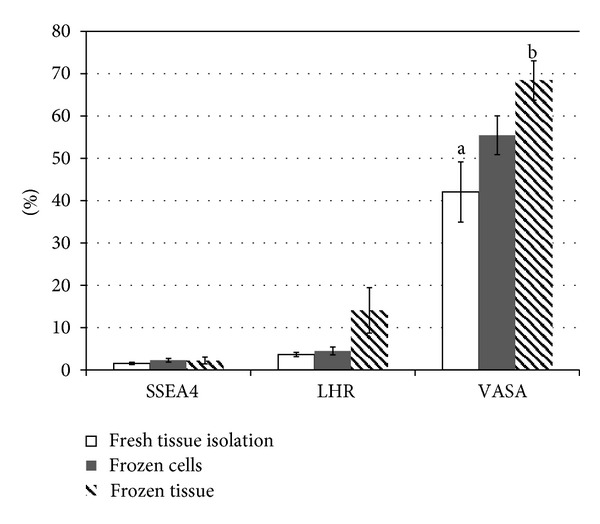
Flow cytometry analysis of the enrichment of cells positive for each marker before and after cryopreservation. The numbers of viable cells positive for each marker were determined by flow cytometric analysis and are expressed as a percentage of the total viable cells in each condition. VASA positive cells were fixed before staining. VASA positive cells were counted without information about which ones were viable or dead. This graph shows that the average percentage of cells positive for each marker is higher after cryopreservation of cells and tissue. Two-sample *t*-test was used for statistical analysis and *P* < 0.05 was considered significant. _ _^ab^ *P* = 0.0188.

Immunolocalization of the cells positive for the specific markers used in this study is presented in Figure 1. Testicular cell isolations are a mixture of the various component cells of the testes, most importantly to fertility preservation are SSCs. The survival of these cells after cryopreservation is the key to fertility preservation or other therapeutic regenerative medicine techniques. The recovery and the percentage of the cells positive for each cell marker before and after cryopreservation are presented in Table 3 and Figure 2, respectively. For every gram of fresh tissue, on average 630,923 viable SSEA4+ cells were recovered. After cell thawing an average of 246,578 of those cells were recovered. This represented a recovery rate of 39.1%. By comparison, when tissue pieces were cryopreserved, thawed, and enzymatically digested, an average of 50.4% of the number of SSEA4+ cells isolated per gram of fresh tissue was recovered—higher by 11.3% (50.4%–39.1%) in absolute terms than when cryopreserving isolated cells. However, only 3 out of the 5 patients showed a higher recovery percentage when cryopreserving tissue rather than isolated cells. Although there is a trend that cryopreserving testicular tissue allows for more survival of the SSCs than cryopreserving isolated cells, the difference was not statistically significant (*P* = 0.4254). 

Interestingly, the recovery rate of SSEA4+ cells was higher than the recovery rate of the total cell population. For cryopreserved cells, the average recovery of SSEA4+ cells was higher by 9.0% in absolute terms. For cryopreserved tissue, recovery rate of SSEA4+ cells was higher by 6.1% in absolute terms compared with the average recovery of the total cell population. The difference in recovery of SSEA4+ cells between cryopreserving cells and cryopreserving tissue was not significant (*P* = 0.14), although 4 out of 5 patients had higher recovery of SSEA4+ cells from cryopreserved tissue. The difference between the recovery of viable SSEA4+ cells isolated before and after cryopreservation of cells was not statistically significant (*P* = 0.1484), as was the difference between fresh tissue isolation and frozen tissue isolation (*P* = 0.5432). Even though cryopreservation leads to the loss of some SSEA4+ cells, it enriches the total population for SSEA4+ cells in the cell suspension. 

Leydig cells produce testosterone and are important for the proper proliferation and differentiation of SSCs into functional gametes [16]. As such, their survival could be important for postcryopreservation maturation of SSCs. An average of 46.8% of Leydig cells as indicated by LHR was recovered after cryopreserving isolated cells. When cryopreserving tissue pieces, on average 138.4% of LHR+ cells were recovered—an indication that more LHR+ cells are able to be isolated by first cryopreserving the tissue than if the LHR+ cells were isolated from fresh tissue. Again, 4 out of the 5 patients had higher recovery of LHR+ cells from cryopreserving tissue compared with cryopreserving cells, although the results were not statistically significant (*P* = 0.1225).

Similar to SSCs, as indicated by SSEA4, cryopreservation enriched the population of supporting Leydig (LHR+) cells in the total cell population. The average recovery of LHR+ cells in the cryopreserved cells was higher by 16.7% in absolute terms than the average recovery of the total cell population. The average number of LHR+ cells isolated from cryopreserved tissue was higher by 94.1% in absolute terms than the recovery of the total cell population from the same tissue. Both cryopreserved cells and tissue had a higher rate of recovery of LHR+ cells than total cell population in 3 out of the 5 patients while at the same time neither enrichment was statistically significant (*P* = 0.4136 and *P* = 0.0743, resp.). The results suggest that LHR+ cells are enriched by both tissue and cell cryopreservation.

VASA is an intracellular transcription factor expressed in all germ cells [17] and was used as an indicator of the survival of the total germ cell population during cryopreservation. Thawed cells isolated from fresh tissue, on average contained 46.4% of the VASA+ cells that were cryopreserved. When tissue was cryopreserved, the number of VASA+ cells isolated per gram of tissue was on average of 65.4% compared with the number of VASA+ cells isolated per gram of fresh tissue. In 3 out of 5 patients, more VASA+ cells were recovered from cryopreserving tissue and in the other two cryopreserving cells preserved more VASA+ cells. However, the greater number of VASA+ cells recovered after tissue cryopreservation was not statistically different from cryopreservation of cells (*P* = 0.0731).

VASA+ cells were enriched when cryopreserving either cells or tissue. For cryopreserved cells, the average recovery rate of the VASA+ cells was higher by 16.3% in absolute terms than the average recovery rate of the total cell population. Similarly, for cryopreserved tissue, the average number of VASA+ cells isolated per gram of tissue was enhanced by 21.1% in absolute terms. Neither enrichment was statistically significant (*P* = 0.2334 and *P* = 0.3052, resp.) (Figure 2).

### 3.4. Sterility Testing

To ensure that there was no contamination of either the cells/tissue or the processing environment sterility testing was performed on the tissue transport PBS, on the cryopreserved cells, the thawed cells, and the air and surfaces in the clean room. For 3 out of the 5 patients, PBS and cells were sent to a CLIA-approved and FDA-registered clinical microbiology laboratory. Each sample of PBS used to transport the tissue was found to be contaminated with several microorganisms. This was likely due to contamination during surgical removal of the tissue. In contrast, all three cryopreserved cell products and thawed cells were completely free from contaminating microorganisms. This indicates that not only the process was aseptic (in that it did not introduce contamination) but our cell processing procedure actually eliminated contamination that was present before the process. Additionally, the processing space where the cell isolation, cryopreservation, and thawing took place was tested for the presence of microbial contamination: samples from both the surfaces and air in the clean room were collected, tested, and found to be free of any viable microorganisms—an indication that the space remained aseptic from outside air and that nothing from the cells/tissue contaminated the working space. 

## 4. Discussion

In the present study, the feasibility of a clinical-grade cryopreservation procedure for human testicular cells and tissue obtained from sexual reassignment patients was studied. Our results clearly show that cryopreservation of human testicular cells and tissue with a protocol compliant with cGTP and some cGMP requirements results in acceptable viability and cell recovery of different testicular cell subpopulations. Also our results clearly demonstrate that human testicular tissue processed and cryopreserved under the cGMP environment described here are free from microorganism and can be used for clinical applications.

Finding a clinical-grade method for preservation of testicular cells or tissue would be very helpful in allowing these cryopreserved biologics to be used for future regenerative medicine applications in humans. Without a validated procedure, regulatory agencies would not allow testicular cells/tissues to be transplanted back into the patient's body. Other methods have shown that it is possible to effectively isolate and/or cryopreserve testicular cells and/or tissues; however, these methods did not comply with cGTP or cGMP regulations or employ clinical-grade reagents/supplies or possess the rigorous documentation that would make the procedures permissible for a clinical study. In our protocol, cGTP and some cGMP conditions were complied with throughout. All 5 tissues were processed using clinical-grade and/or cGMP grade materials and reagents. All the tools and reagents that directly came into contact with the cells and tissue were sterile. The equipment was properly validated, the procedures codified in SOPs, proficiency assessed and ascertained, and the critical information for the process, equipment, and reagents/supplies (vendor, catalog numbers, lot numbers, serial numbers, expiration dates, certificates of sterility, conformity, and analysis) were documented. 

Two major components of our freezing medium are DMSO and Dextran (a disaccharide). Both DMSO and disaccharides (e.g., sucrose) have been widely used for cryopreservation of testicular cells [11] and tissue [18]. In addition, the DMSO-Dextran cryopreservative has been frequently used for and validated in human cord blood [26], bone marrow [27] and peripheral blood stem cell freezing and other clinical applications. In this protocol fetal calf serum (FCS), used in previous studies, was replaced with HSA. FCS use should be avoided for clinical protocols because its animal origin increases the risk of contaminating the cells and/or tissue with bovine blood-borne pathogens. On the other hand, HSA is approved for human use and has been successfully used in other cryopreservation protocols including peripheral blood mononuclear cells [28] and ovarian tissue and follicles [29].

For cryopreservation, in addition to controlled rate freezing, we also tested manual freezing and found no effect on cell viability after thawing (data not shown). This is in agreement with a previous study in a bovine model [10] and our own unpublished data in the mouse and primate models indicating that manual freezing provides similar freezing conditions to the controlled rate freezing. However, controlled-rate freezing is preferred to ensure more reproducible conditions. Controlled-rate freezing also provides the ability to perform the cryopreservation in properly validated equipment with documentation detailing the cryopreservation process—in compliance with cGMP regulations. Our results give credence for the first time that this clinically applicable procedure can be used to effectively produce cells or tissue that are safe for potential use in humans.

We found that cryopreserving tissue may be a better method for preserving testicular cells using this clinical-grade procedure—especially regarding the viability of those cells. It should be noted that in this population of hormone-treated patients, the viable cell number recovery was not statistically significant, with the variance of results possibly affected by the variability of hormone treatment. In all categories, on average, cryopreserving tissue yielded more cells after cryopreservation than cryopreserving cells, though none of the comparisons yielded statistically significant comparisons in this sample size of variable tissue except viability. Of particular interest in the testicular cell population are the SSCs, represented in this and other studies by SSEA4+ cells [14, 15]. These cells have the potential to be transplanted back into the body to restore fertility, to be terminally differentiated into sperm for assisted reproductive techniques, or to be differentiated into different cell types for other regenerative medicine applications. Cryopreservation of testicular tissue generated greater recovery of SSEA4+ cells in this study. Enrichment of SSCs due to cryopreservation and thawing has also been reported by other investigators [30].

The same was true for the other cell population investigated in this study. Leydig cells are responsible for production of testosterone and maturation of SSCs into functional gametes. Cryopreserving both cells and tissue enriched the total cell population for cells positive for LHR (a Leydig cell marker) but when cryopreserving tissue, the LHR+ cells were even more enriched after cryopreservation and thawing. The fact that Leydig cells survive cryopreservation so effectively indicated that these cells perhaps are more resistant to cryodamage and could support the germ cells after cryopreservation. In an effort to preserve human testicular tissue, it has been shown that Leydig cells are more resistant to cryopreservation compared to spermatogenic cells [12]. This is important as these cells could play a key role in maturation of SSCs to functional sperm. Further experiments are needed to investigate this hypothesis. Among all cell types present in the adult testes, more differentiated spermatogenic cells including spermatocytes, spermatids, and spermatozoa are the most abundant cell types in seminiferous tubules and perhaps the most susceptible to cryodamage. We used VASA as a general germ cell marker in this study and the majority of the VASA positive cells in the testes are the advanced germ cells. Surprisingly, VASA positive cells were also enriched after cryopreservation. This might be due to the fact that hormone treatment in these patients has eliminated the majority of the advanced germ cells and, in fact, what is left from the VASA positive cells are the early-stage germ cells including SSCs and differentiating spermatogonia. Deleterious effects of steroid hormones, normally used in sexual reassignment patients, on spermatogenesis has been reported [31]. Similar to SSCs and Leydig cells, VASA positive cells were less susceptible to damage when they were cryopreserved as part of whole tissue. In general, all three cell types that have been examined in this study were better preserved when the tissue was frozen as compared to cryopreservation in cell suspension.

There are many considerations that go into determining if cryopreservation of testicular cells or tissue is the most appropriate. The quality of the cells that result from either cell or tissue freezing is important—as measured here by the viability and the number of cells isolated (both the total viable population and the survival/enrichment of the SSCs, Leydig cells, and germ cells). Other considerations are also taken into account: cryopreservation of testicular tissue requires less time and effort, is less expensive, and requires less equipment. On the other hand, cryopreserving cells isolated from fresh tissue has its advantages. First, more information is known about the cells—such as the quality of the cells as explained above. Secondly, the process of isolation and cryopreserving the cells eliminates any contamination found in the transport PBS before processing. Thirdly, it is much easier to perform quality control and stability testing on the cryopresevered cells. Finally, the cryopreserved cells can be tested for sterility. Sterility testing in particular takes several weeks to obtain using the methods currently employed in CLIA-approved laboratories as indicated in this study. If testicular tissue is cryopreserved the results of sterility would not be obtained until far after the cells would be isolated and transplanted back into the body for fertility preservation. For these reasons, cryopreservation of testicular cells isolated from fresh tissue is favorable over cryopreservation of tissue pieces (Table 4).

To the best of our knowledge, this is the first study showing that testes of sexual reassignment patients contain sufficient amounts of SSCs, Leydig cells, and germ cells that can be effectively cryopreserved. However, there were some variations between tissues from different patients especially in the number of viable cells isolated per gram of tissue. In this respect, it can be speculated that these differences might be due to the effect and length of treatment of the estrogen and other hormones used in the sexual reassignment procedure, their effect on spermatogenesis, and individual patient susceptibility. The more estrogen is used and the longer it is administered, the more spermatogenic cells will be eradicated, the emptier the tubules will be, and the more volume of the seminiferous tubules will be filled with fluid rather than cells. This leads to an overall decline in the number of cells isolated per gram of tissue. Therefore, patients with less treatment will likely be less affected, with more cells isolated per gram of tissue. Because these patients had varying amount of estrogen treatment, it is more important to look at what percent of cryopreserved cells were recovered from cell and tissue cryopreservation to understand the effectiveness of the cryopreservation procedure. In that regard, cryopreserving tissue has a nonstatistically significant edge. In the future, we hope to expand our understanding of testicular tissue freezing and the applicability of either cryopreserved cells or tissue for use in a clinical setting. First, repeating this study on adult human testicular tissue that has not undergone hormone treatment. In particular, repeating this experiment on testicular tissue from prepubertal boys will provide a clinically applicable procedure that would give young patients the possibility of restoring their fertility. Including patients with other conditions or therapies that threaten male fertility will expand the applicability of this procedure.

The potential application of cryopreserved testicular cells or tissue needs investigation to make clinical use of this material. A procedure for reimplantation of the testicular cells into a healthy testis needs to be developed. For cancer-survival patients, that kind of procedure presents a risk of reintroduction of cancer to their bodies. Those patients and others such as males who have completed the male-to-female sexual reassignment surgery, testicular torsion or varicocele patients for whom fertility restoration is not possible could use their cryopreserved cells for assisted reproductive techniques. A study describing the methods and effectiveness of these applications, including possible maturation of SSCs into functional gametes, would allow for the use of their cryopreserved material. Additionally, the results of this study would be improved by comparing controlled rate freezing and vitrification of human testicular tissue. Vitrification has shown a better success in cryopreservation of ovarian tissue [32, 33]. Similarly, vitrification has proven to preserve survival, development, integrity, and functionality of prepubertal testicular tissue in mouse [34], nonhuman primate [35] and human [36]. Therefore, in future studies we would like to compare the effectiveness of slow freezing and vitrification on cryopreservation of testicular tissue obtained from hormone treated as well as untreated patients.

## 5. Conclusions

A clinically applicable method successfully cryopreserved human testicular cells and tissue. We found for the first time that testicular tissue and cells from patients undergoing sexual reassignment can be successfully cryopreserved—and important cell populations can be enriched by the cryopreservation process. Sterility tests show that the cryopreserved cells and the thawed cells processed by our protocols under cGTP and some cGMP conditions were free from any microbial contamination. Cryopreservation of testicular cells seems to be more clinically applicable than cryopreservation of testicular tissue.

## Figures and Tables

**Table 1:** Viability of cells before and after cryopreservation.

Patient	Viability of cells		
	Fresh cells	Frozen cells	Frozen tissue
1	84.9%	38.1%	70.1%
2	91.9%	64.1%	81.8%
3	93.0%	53.8%	68.9%
4	90.6%	53.3%	75.9%
5	90.2%	52.8%	73.5%

Average	90.1%	52.4%^a^	74.0%^b^
SEM	1.3%	3.9%	2.2%

The viability of each cell population was determined by dividing the number of viable cells counted with the number of total cells counted (viable + dead). Two sample *t* test was used for statistical analysis and *P* < 0.05 was considered as significant. ^ab^ *P* = 0.0019.

**Table 2:** Number of viable cells per gram of tissue and recovery of cryopreserved cells.

Patient	Viable cells per gram of tissue			Percent Recovery	
	Fresh cells	Frozen cells	Frozen tissue	Frozen cells	Frozen tissue
1	15,300,000	4,057,018	6,630,259	26.5%	43.3%
2	42,800,000	13,312,147	32,958,482	31.1%	77.0%
3	26,900,000	4,114,322	17,889,704	15.3%	66.5%
4	68,624,368	21,006,944	11,224,466	30.6%	16.4%
5	58,877,485	27,549,020	10,704,884	46.8%	18.2%

Average	42,500,371	14,007,890	15,881,559	33.0%	37.4%
SEM	9,313,258	4,390,103	4,390,059	4.8%	11.7%

The number of cells calculated per gram of tissue is compared between isolating cells from fresh tissue, cryopreserved cells, and cryopreserved tissue. For fresh cells, the weight of the tissue of each enzymatic digestion was determined. For frozen cells, the number of cells recovered from each frozen vial was used to calculate how many cells would have been recovered had the cells from 1 gram of tissue been frozen. For frozen tissue, the number of cells isolated from each piece after thawing was used in conjunction with the weight of the tissue before freezing. For all three, only viable cells were used for the calculations. Cell recovery was calculated by dividing the number of viable cells per gram of tissue from either the cryopreserved cell or cryopreserved tissue with the viable cells from fresh tissue cell isolation.

**Table 3:** Recovery of viable cells positive for cell marker per gram of tissue.

Patient	SSEA4+			LHR+			VASA+		
	Fresh cells	Frozen cells	Frozen tissue	Fresh cells	Frozen cells	Frozen tissue	Fresh cells	Frozen cells	Frozen tissue
1	382,500	133,392	126,144	459,000	222,571	523,697	10,128,600	2,894,214	5,642,505
2	642,000	271,081	540,099	1,883,200	378,955	5,003,870	8,089,200	5,820,763	24,075,066
3	215,200	141,845	215,335	1,102,900	67,546	1,069,848	10,733,100	1,939,856	11,733,700
4	829,602	259,954	108,069	3,402,562	1,201,910	644,282	28,455,915	11,992,882	6,595,656
5	1,085,311	426,618	599,564	1,141,806	1,865,490	3,814,563	25,813,056	15,991,324	6,356,519

Average	630,923	246,578	317,842	1,597,894	747,294	2,211,252	16,643,974	7,727,808	10,880,689
SEM	146,814	50,549	99,337	477,708	323,267	872,536	4,096,440	2,567,434	3,288,223
Percent recovery	N/A	39.1%	50.4%	N/A	46.8%	138.4%	N/A	46.4%	65.4%

The number of cells positive for each marker per gram of tissue isolated from fresh tissue cell isolation and recovered from either frozen cell or frozen tissue. Percent Recovery was calculated by dividing the average after cryopreservation by the average before cryopreservation. No significant differences were observed.

**Table 4:** Pros and cons of cryopreservation of testicular cells and tissue for clinical application.

Material to Freeze	Pros	Cons
Tissue	(1) Requires less time and effort. (2) Less expensive. (3) Requires fewer equipment to keep validated.	(1) Cells have to be isolated first before transplantation or other procedure. (2) No information about the sterility of the cell product can be acquired at the time of transplantation. (3) No information about the cellular composition is collected. (4) No quality control and stability test can be done.

Cells	(1) A complete profile of the viability, sterility, and cellular composition has been collected before cryopreservation. (2) Small samples of cell product can be used for quality control and stability testing during long-term storage. (3) The cell product with the known quality can be safely stored and sent to the clinic prior transplantation.	(1) Requires more time and effort for cell dissociation. (2) More expensive. (3) Has to be done in a cGTP compliant cell processing facility.
